# Change management with empowerment of nursing staff to reduce urinary catheter use

**DOI:** 10.1186/2047-2994-4-S1-P217

**Published:** 2015-06-16

**Authors:** N Bartlomé, A Conen, E Bucheli, S Schirlo, CA Fux

**Affiliations:** 1 Clinic for Infectious Diseases and Hospital Hygiene, Kantonsspital Aarau, Aarau, Switzerland; 2 Medical Nursing staff, Kantonsspital Aarau, Aarau, Switzerland

## Introduction

Catheter-associated urinary tract infections (CAUTI) are the most common nosocomial infections.

## Objectives

We used a multi-modal interdisciplinary intervention to reduce CAUTI with three key elements: stringent indications for UC insertion, shifting the task to decide on urinary catheter (UC) removal from physicians to nurses and an automatic electronic alert for catheter removal as key elements.

## Methods

Non-randomized intervention study.

## Patients and methods

We included all patients with a newly inserted UC at any time during hospitalization. The 13-months study comprised a baseline and 2 intervention phases. Clinical endpoints included the number of catheter days per 1’000 hospital days, the duration of catheterization as well as the rates of inserted catheters and CAUTI. Process endpoints compared changes in attitudes and knowledge about UC and CAUTI between physicians and nurses.

## Results

Overall, 9’306 patients were screened for newly inserted UC, of them 513 (5.5%) were included. In these 513 patients, the number of catheter days was reduced from 88.5 to 31.9 days per 1’000 hospital days (p<0.001) with a mean and median reduction of the duration of catheterization from 7.2 to 3.8 and 5 to 3 days, respectively (p<0.001). The number of overall CAUTI was reduced with a risk ratio of 0.31 (95% CI 0.19-0.49) per 1’000 hospital days and of 0.35 (95% CI 0.21-0.57) per 1’000 hospital admissions. Significant changes in task-shifting from physicians to nurses and in indications for UC were documented.

## Conclusion

Behavioral changes including the empowerment of nurses resulted in significant reductions in the rate and duration of urinary catheterization as well as CAUTI.

## Disclosure of interest

None declared.

## References

[B1] Klevans RM Edwards JR Richard CL Estimating health care-associated infections and deaths in US hospital Pub Health Report 2007 122 160 166 10.1177/003335490712200205 PMC1820440 17357358

[B2] Kilgore ML Ghosh K Beavers CM The costs of nosocomial infections Med Care 2008 46 101 4 18162862 10.1097/MLR.0b013e3181468991

[B3] Graves N Tong E Mortin AP Factors associated with health care-acquired urinary tract infection Am J Infect Control 2007 35 6 387 392 17660009 10.1016/j.ajic.2006.09.006

[B4] Egger M Balmer F Reduction of urinary catheter use and prescription of antibiotics for asymptomatic bacteriuria in hospitalised patients in internal medicine Swiss Med Wkly 2013 143 w 13796 10.4414/smw.2013.13796 23740332

